# Could Lipoprotein Lipase Play a Role in Alzheimer's Disease?

**DOI:** 10.1100/tsw.2004.111

**Published:** 2004-07-20

**Authors:** Jean-Francois Blain, Judes Poirier

**Affiliations:** Douglas Hospital Research Center, Verdun, Quebec, Canada; ^2^ McGill Center for Studies in Aging, 6825 LaSalle Blvd, Verdun, Quebec, H4H 1R3, Canada

**Keywords:** lipoprotein lipase (LPL), cholesterol, lipids, Alzheimer’s disease (AD), synaptic remodeling, proteoglycans, amyloid

## Abstract

This paper reviews recent literature on the role of lipoprotein lipase in the central nervous system with a focus on its recently described role in synaptic remodeling. This novel role could have implication for Alzheimer's disease treatment.

In 2000, there were an estimated 18 million persons worldwide with dementia, with more than half suffering from Alzheimer's disease (AD). Considering the aging of the population and a projected increase in survival, this number is predicted to reach 37 million by 2025.

It is now well established that the causes for familial AD (10–15% of all AD cases) are associated with an increased production of a peptide called beta-amyloid (Aβ), resulting from mutations in the genes coding for either the amyloid precursor protein (APP)[1,2,3], presenilin-1[4,5], or presenilin-2[6]. It was also shown that a polymorphism in the apolipoprotein (apo) E gene, giving rise to an isoform called apoE4, is strongly associated to both familial and sporadic (common form) AD. ApoE4, a common transporter of lipid in the brain and in periphery, is now recognized as the major risk factor for the development of late-onset sporadic and familial AD[7,8,9].

ApoE in the brain regulates the transport and distribution of cholesterol and lipids from the astrocytes, where they are stored, to neurons that are in need of these constituents for membrane synthesis and reinnervation purposes. Several studies reported close association between cholesterol metabolism, Aβ generation, and amyloid deposition[10,11,12,13,14]. Thus, it is not surprising to see cholesterol homeostasis become a major target for potential therapy in AD[15,16,17].

In a recent report[18], we described a potentially new function for lipoprotein lipase (LPL) during synaptic remodeling in the mouse adult brain. In periphery, LPL is known to hydrolyze triglycerides, but also helps to regulate the internalization of lipoproteins in different cell types. This protein is also interesting from an AD point of view as it interacts with brain lipoproteins and binds to their receptors, modulating cholesterol homeostasis in neuronal cells.

LPL deficiency has been linked to specific neurological disorders, supporting the notion of a central role in brain functions[19,20]. While being expressed throughout the brain, LPL was shown to have its highest levels in the hippocampus[21,22,23]. Its activity increased during brain development with a peak at 10 days postnatal, followed by a progressive decline in adulthood[21]. To be catalytically active, LPL requires the presence of significant amounts of apoCII, a cofactor relatively absent from the brain parenchyma[24,25,26,27]. On the other hand, it was shown to modulate, in a cofactor-independent manner, the binding and internalization of lipoprotein particles[28,29]. LPL is involved in the transfer of vitamin E to tissues[30] and it mediates the internalization of cholesteryl ester-rich HDL particles[31,32,33]. These particles exhibit properties similar to the HDL particles found in the CSF and the brain[34,35]. LPL is known to bind to the different members of the low-density lipoprotein receptor (LDLR) family[36,37,38,39,40], but its major binding site is on the heparan sulfate proteoglycans (HSPGs) found at the surface of the plasma membrane[41,42,43]. In the peripheral sciatic nerve crush, an increase in LPL was observed and it was proposed to act as a recycling system for the lipid breakdown products[44]. It was also shown to induce neurite outgrowth of sympathetic neurons through LRP-mediated endocytosis[45]. In relation to AD, LPL was found to accumulate in senile plaques[46] and a specific genetic polymorphism was shown to be associated with the disease incidence in clinically diagnosed AD subjects[47]. Recently, three independent studies failed to replicate the reported association in different cohorts of subjects[48,49,50].

Using a mouse model of hippocampal deafferentation that mimics some of the pathological features found in AD, we showed that LPL mRNA and protein levels were upregulated during the degenerative phase, with a peak at 2 days postlesion. Levels were back to basal levels shortly after the reinnervation phase. This led us to propose a role for LPL in the recycling of the lipids and cholesterol released from the degenerating terminals toward the reinnervating neurons. Interestingly, Paradis et al. also showed increased LPL expression early after brain damage using a focal ischemia model[23] as well as an increase in neurite outgrowth of LPL-transfected cells exposed to VLDL[51].

As LPL is known to bind to several members of the LDL receptor family, we examined the expressions of LPL target protein as a function of deafferentation and reinnervation in the lesioned animals. In addition to the classic LDL receptor, we also examined the LRP and apoER2 expression as well as that of syndecan-4, a glia-specific HSPG. LRP and apoER2 levels are not affected by the deafferentation process, whereas the LDLR expression is somewhat downregulated 6 days after the lesion. On the other hand, the proteoglycan syndecan-4 was shown to be upregulated at 2 days postlesion in astrocytes, coinciding with the peak expression of LPL in the deafferented hippocampus. It is thus tempting to propose that LPL-rich lipoprotein complexes present in the extracellular space interact with the cell-surface syndecan-4 on astrocytes to permit the functional transfer of cholesterol from storage organelles toward the cell surface where lipoprotein complexes are anchored to the membrane. ApoE, whose expression level peaks a few days later, would subsequently be added to the lipoprotein structure. The cholesterol-rich complex could then diffuse toward nearby neuronal cells expressing apoE receptors belonging to the LDLR family. The complex would then be internalized and the cholesterol released intracellularly for membrane assembly purposes.

To summarize, we reported the first evidence that LPL is actively involved in synaptic remodeling in the adult CNS following deafferentation and that glial syndecan-4 is an integral part of this process. The members of the syndecan family as well as LPL were shown to be present in senile plaques and neurofibrillary tangles in the AD brain[52,53]. The recent development of experimental therapies based on the binding properties of Aβ to the glycosaminoglycan side chains of HSPGs such as syndecan-4[54,55] is consistent with the role we propose for it in remodeling.

## ACKNOWLEDGMENTS

The authors would like to acknowledge the financial support of the Alzheimer's Society of Canada and the Canadian Institute of Health Research.
